# Six Myths and Misconceptions about Essential Tremor

**DOI:** 10.5334/tohm.948

**Published:** 2024-09-25

**Authors:** Elan D. Louis

**Affiliations:** 1 Department of Neurology, University of Texas Southwestern Medical Center, Dallas, Texas, USA; 2 Peter O’Donnell Jr. Brain Institute, University of Texas Southwestern Medical Center, Dallas, Texas, USA

**Keywords:** Essential tremor, myths, misconceptions, clinical, classification, mortality

## Abstract

There are myths and misperceptions about most human diseases, and neurological diseases are no exception. In many instances, myths and misconceptions reflect what is no more than the collective failure of the field to catch up with the state of the science in that field. Hence, one may perhaps refer to these as “lags” rather than myths. As the field of medicine attempts to be evidence-based, it is best to remain true to published data and the state of the science. In this paper, I review six myths and misconceptions about ET. Myth 1 relates to the natural history and prognosis of ET. Myths 2 and 3 relate to the biological basis of ET, whereas myths 4 and 5 relate to the expression of the core clinical feature of ET. Finally, myth 6 focuses on the issue of disease classification. The myths are as follows: *Myth 1:* “ET is not associated with a shorter life expectancy”. *Myth 2:* “The pathophysiology of ET remains unclear”. *Myth 3:* “There have also been studies that do not show any cerebellar degeneration”. *Myth 4:* “ET is a postural or a kinetic tremor”. *Myth 5:* “Action tremor in ET is usually bilateral and symmetric”. *Myth 6:* “ET plus”. As neurologists, we are not ignorant of feedback loops. A regular review of facts should help to frame one’s output. As such, one’s formulations and output will be firmly grounded in data.

## Introduction

There are myths and misperceptions about most human diseases, and neurological diseases are no exception. In fact, there is a literature that may be traced back to the 1970s, which explores a number of myths and misperceptions (“neuromythologies”) in our field [1,2,3,4].

In many instances, myths and misconceptions reflect what is no more than the collective failure of the field to catch up with the state of its own science, and one should perhaps refer to these as “lags” rather than myths.

Essential tremor (ET) is not exempt from these myths and misconceptions, and these have a way of being stated and then re-stated in research articles and review articles. Their incorporation in review articles is particularly problematic as these types of articles tend to be both highly read and cited, and if their information is not accurate, this can easily result in an amplification of misinformation.

With respect to our understanding of the clinical and biological features of ET, there is a rich literature, reflecting a record of scientific inquiry and resultant data over many decades. As the field of medicine attempts to be as evidence-based as possible, it is best to remain true to the data. In this paper, I review six misconceptions about ET. Myth 1 relates to the natural history and prognosis of ET. Myths 2 and 3 relate to the biological basis of ET, whereas myths 4 and 5 relate to the expression of the core clinical feature of ET. Finally, myth 6 focuses on the issue of disease classification.

## Myth 1: “ET is not associated with a shorter life expectancy”

One often-made statement is that ET is not associated with shorter life expectancy [5]. In fact, studies of risk of mortality in ET are scarce. There has only been one prospective, longitudinal population-based study of mortality in ET [6]. In that study, participants with ET and controls were studied in three communities in central Spain [6]. All participants were evaluated at baseline and at follow-up 3 years later [6]. The mean baseline age was 73.5 ± 6.4 years. During the study period, there were 33 (16.4%) deaths among 201 ET cases and 465 (13.9%) deaths among 3,337 controls [6]. In an unadjusted Cox model, the risk of mortality was increased in ET (relative risk [RR] = 1.59, 95% confidence interval [CI] = 1.11–2.27, p = 0.01) [6]. In a Cox model that adjusted for baseline age, gender, educational category, current ethanol drinking, use of antidepressant medication, and community, the RR = 1.45 (95% CI = 1.01–2.08, p = 0.04) [6]. In an adjusted Cox model restricted to participants who had had longer (i.e., more than 3 years) follow-up, RR = 4.69 (95% CI = 2.18–10.07, p = 0.001) [6].

Historically, there have been surprisingly few data on risk of mortality in ET. In the early 1900s, one investigator reported that 1 (6.9%) in every 14.5 of his ET patients was 80 years of age, compared to 1 (0.9%) in every 116 people in the general population of France [7]. In a more recent analysis, the investigator assessed data on ages of parents of patients with ET [8]. Parents who were reported to have had tremor lived a median of 8 years longer than did parents who reportedly had not had tremor [8]. One interpretation is that tremor confers longevity; however, the more likely explanation is that the incidence of ET increases with age [9,10]; as such, older individuals are more likely to develop ET than are younger individuals. Stated in another manner, living to an older age increases the likelihood of developing ET, rather than the converse, that having ET increases one’s lifespan [6].

There is a single study that is somewhat comparable to the Spanish study [6] because both were longitudinal studies. However, that study was retrospective rather than prospective and it used a historical control group rather than controls who were enrolled in the same study as ET cases [9]. Survival among ET cases was reported to be comparable to the expected survival for persons of similar age and sex from the West North Central region of the United States (i.e., the historical control group) [9]. However, the investigators did not formally study time to event outcomes using Cox proportional hazards models, and as such, could not adjust for variable lengths of follow-up and a host of demographic and clinical factors that likely contributed to mortality in their study groups [9]. Furthermore, the participants were quite young – the mean age at inception was 58 years and the mean length of follow-up was 9.7 years, indicating that many of the cases were not followed into advanced age [9], when risk of mortality is highest [6].

In summary, in the one longitudinal, prospective, population-based study that compared patients with ET to their counterparts without ET, the risk of mortality in ET was increased by 45% (RR = 1.45) and, in an analysis that was restricted to persons with longer follow-up, the risk of mortality in ET was further increased (RR = 4.69) [6]. A limitation of the current literature is the relative dearth of studies. While confirmatory studies are needed, the above are the most robust available data, and they are based on a strong study design. Stating that ET is not associated with a shorter lifespan, therefore, is an outdated formulation.

A demonstrated increased risk of mortality in ET is not a surprising one. ET is a progressive disease [11] and patients with severe tremor have difficulty performing activities of daily living [12]. The presence of ataxia and difficulty ambulating [13] as well as cognitive impairment and dementia [14], in concert with recent observations of degenerative pathologies in postmortem studies of ET brains [15], provide a framework for the observed increased risk of mortality in ET. Indeed, a study of 141 elders with ET demonstrated that cognitive impairment and gait and balance difficulty were each associated with increased risk of mortality in this disease [16].

## Myth 2: “The pathophysiology of ET remains unclear”

Statements to the effect that the pathophysiology of ET remains unclear are often made [5,17], leading to superficial and often-truncated discussions about disease mechanisms. While such statements may have been appropriate twenty or thirty years ago when postmortem studies were virtually nonexistent [18], with the subsequent publication of autopsy series in excess of 100 and 200 ET brains [15,19], this is no longer a fitting or valid statement. Of course, the full details regarding the pathophysiology of ET are not known, but these full details are not known about any of the neurodegenerative diseases, whether this is Parkinson’s disease (PD), Alzheimer’s disease (AD) or Huntington’s disease. However, the broad pathophysiology of ET is understood. The basis for ET is a breakdown in normal anatomy and physiology along the cerebellar-thalamic-cortical pathway [20,21,22,23]. A major component of the disease, and a likely starting point, is degenerative pathology in the cerebellar cortex [15]. What is not fully understood is the precise physiological mechanism through which the degenerative pathology in the cerebellum leads downstream, through the dentate nucleus, to action tremor [20,24,25]. The role of the inferior olivary nucleus, which for many years was thought to be the primary generator of tremor, is not well understood either [26], although it is still speculated by some that it may play a role in the pathophysiology of ET. It is also likely that ET is a family of diseases rather than a single disease, and that multiple etiologies and resultant molecular mechanisms converge from different starting points on the above-mentioned cerebellar-thalamic-cortical pathophysiological pathway [27,28].

## Myth 3: “There have also been studies that do not show any cerebellar degeneration”

There are now more than 70 postmortem studies, spanning a period from 2005 to present, demonstrating degenerative changes in the ET cerebellar cortex [15,20,29]. These studies have demonstrated in fine detail a large number of degenerative changes in the Purkinje cell population as well as neighboring neuronal populations, with more than thirty such changes carefully documented [15,18,20,29,30,31]. All of these studies are controlled, thereby indicating that these changes are not age-related changes, but are disease-linked [15,18,20,29,30,31]. These changes are not haphazard, and an ordered cascade of cellular-degenerative events has been proposed [20]. The features of degeneration in the ET cerebellum are of the type seen in other disorders of cerebellar degeneration – falling well within the spectrum of what is seen in those disorders [15,32]. These degenerative changes have been reported by a several research groups, including those associated with the Essential Tremor Centralized Brain Repository [15,18,20,29,30,31], as well as investigators in Greece [33,34] and Canada [35,36]. From a clinicopathological correlative vantage point, key neuropathological features of ET lateralize with clinical features [37]. As such, a revealing study demonstrated that the arm with the more severe action tremor was ipsilateral to the side of the cerebellum with the more severe pathology [37]. A recent analysis using data from a small number of these degenerative cerebellar changes demonstrated that it was possible to distinguish ET and control brains with approximately 95% sensitivity and specificity and, as such, taking a decided step towards tissue-based diagnostic criteria for ET [38].

With this large backdrop, there is a single study, from a decade ago, that (1) was not able to reproduce the finding of *one* of these pathological changes, and importantly, (2) did not assess any of the large number of other changes [39]. The study as well as its null result have been critiqued repeatedly, and methodological features of the study make its validity questionable [20,40,41,42] (especially see extensive discussion in “PC death” section of Louis and Faust [20]). Regardless, the statement that “there have also been studies that do not show *any cerebellar degeneration”* is erroneous [21]. The correct characterization would be most appropriate in footnote form – in contrast to the extensive collection of data arising from several groups of numerous degenerative changes in the ET cerebellum, there is one study, with considerable methodological problems, that was unable to confirm a single one of these changes. That one study did not examine any other changes.

## Myth 4: “ET is a postural or a kinetic tremor”

With respect to upper limb tremor, it is commonly written that the tremor of ET is either a postural or a kinetic tremor [43]. In other words, patients may have one *or* the other. One conceptual extension of this is that isolated postural tremor is consistent with ET. This is a statement that has somehow become ingrained in our literature [43,44,45,46,47,48]. A more factually-based statement is that patients with ET often have both kinetic as well as postural tremor. However, several studies have documented that in the vast majority of patients, kinetic tremor is more marked than postural tremor, and in a large minority, is of considerably greater amplitude [49,50]. The presence of isolated postural tremor is debatable. Therefore, the more valid statement is that patients with ET predominantly have kinetic tremor, and that many may also have postural tremor, although this is of lesser amplitude, in general, and not an isolated feature of ET. A shorthand way of writing this is that the predominant clinical feature of ET is kinetic tremor.

## Myth 5: “Action tremor in ET is usually bilateral and symmetric”

A statement that is commonly made in the literature is that the upper limb tremor in ET is symmetric [51]. This statement may be found threaded throughout the literature [52,53,54,55,56,57]. This is not a universal statement, as numerous authors correctly point out that the tremor is generally somewhat asymmetric, and clinicians who carefully examine their ET patients will notice that the tremor is not symmetric in the upper limbs, and that tremor in one limb, as a general rule, is worse than on the contralateral side. The published data on tremor asymmetry are surprisingly sparse – that is, a limitation of the relevant literature is the dearth of that literature. Nonetheless, there are published data that are of value. A study of 54 ET cases in New York assessed tremor severity using several methods [58]. First, patients underwent a tremor examination, during which the severity of kinetic and postural tremors in the arms were assessed during six tasks rated using a 0 to 3 clinical rating scale. Fourteen patients also underwent quantitative computerized tremor analysis. The mean side-to-side difference in clinical ratings for each of the 6 tasks was 0.54 of 3 points, which represented a 1.32-fold difference between sides [58]. During quantitative computerized tremor analysis, there was a 1.71-fold mean difference between tremor amplitudes in the dominant and nondominant sides [58]. The study concluded that small to moderate differences between sides were common in ET, and that mild asymmetry is a fundamental property of ET [58]. Beyond these data, there are studies that report group-level data on tremor severity in the dominant and non-dominant arms [59,60], but not individual-level data that gauge tremor asymmetry within individuals with ET.

## Myth 6: “ET plus”

ET patients may exhibit a range of disease features apart from kinetic tremor. Not all patients exhibit all features; hence, there is heterogeneity across patients. One natural question that arises from this observation is whether ET is a single entity or several entities. If ET is more than one entity, then how does one subdivide cases? The term “ET plus” was coined in 2018 at a consensus conference, and in a simplistic way, based solely on clinical manifestations, proposed to subclassify ET cases into two subgroups, with one group having only action tremor and no associated features and the other group having both. One misunderstanding that has arisen with the passage of time is the notion that ET plus is not ET, when in fact it was proposed as a subcategory of ET – that is ET was divided into two groups, both with the names “ET”. There are numerous problems with this proposed division scheme, and these are as follows: (1) it is a purely clinical categorization, with no other basis [61], (2) the division is into the “haves” vs. the “have nots” (i.e., simple binary) rather than a well thought-through division scheme based on a true understanding of subgroupings of clinical signs that would allow for a more clinically and biologically-based division point, (3) the two putative subgroups do not differ in important clinical respects (therapeutic response phenotype [e.g., response to DBS surgery] [62,63], rate of progression) [61], or in terms of electrophysiological properties of the tremor [64], (4) the two putative subgroups do not differ with respect to etiological factors (genes, environmental risk factors) [61,65], (5) the two putative subgroups do not differ with respect to underlying disease pathology – a postmortem study of 24 ET and 26 ET plus brains assessed 14 quantitative metrics of cerebellar pathology – there were no between-group differences [66], (6) the two putative subgroups themselves are not stable designations – in a prospective, longitudinal cohort study, for example, of 172 ET cases who received a diagnosis of ET plus at one or more time intervals, the diagnosis was unstable (e.g., with reversion) in 62 (36.0%) [67], (7) the two putative subgroups seem to largely be proxies for disease duration (i.e., ET plus seems to represent a disease stage rather than a distinct disease subtype or disease classification) [61,68], (8) the ET plus definition does not include quantifiable metrics to gauge whether a sign (e.g., cognitive impairment, gait impairment) is present or absent, leaving this up to the subjective assessment of the clinician [61], (9) the term “questionable dystonia” is subjective and difficult to comprehend and it overlooks “non-questionable dystonia” [69], (10) the term introduces unnecessary complications for the effective performance of a range of research studies, including prevalence and incidence studies, longitudinal studies, genetic studies and experimental therapeutics. [61,70], and (11) other “plus” categories in movement disorders have failed [61].

Despite these numerous shortcomings, the term ET plus continues to be used in literature [64,71,72,73].

Heterogeneity in ET certainly exists, but the desire to use observed differences across cases to subdivide them should create division schemes based on meaningful clinical differences as well as observable differences in natural history, prognosis, therapeutic response and underlying biology. A scheme that fails on so many of these accounts has no merit. This is understood by many experts. As a result, the use of the term ET plus has been called into question, as the term, which was initially meant as no more than a temporary label [74], is one that is not backed by sound scientific evidence [61,69,70,75,76,77,78,79] It has been suggested, in fact, that this term be abandoned [76].

Hence, the notion that ET plus has some reality and that there are patients with ET plus is a myth. The reality is that patients with ET are heterogeneous and we have as yet not determined a reasonable and scientifically credible manner by which to subdivide them. There is considerable work for future study groups, beginning with the collection of relevant data in well-designed studies, the critical appraisal of those data, and only if supported by the data, the creation of new constructs.

## Summary

As neurologists, we are not ignorant of feedback loops. Adjustments and corrections come through a regular return to facts, assessing the primary data, and having one’s formulations firmly grounded in these. Here I discussed, in brief, six common myths and misconceptions about ET. Advancing our understanding about a disease process becomes a somewhat futile effort if the discussion that evolves around that disease takes a step backwards and does not make use of published data.
